# Domestic Violence and Mental Health During the COVID-19 Pandemic in Bangladesh

**DOI:** 10.2196/24624

**Published:** 2021-09-13

**Authors:** Tanjir Rashid Soron, Md Ashiqur Rahman Ashiq, Marzia Al-Hakeem, Zaid Farzan Chowdhury, Helal Uddin Ahmed, Chaman Afrooz Chowdhury

**Affiliations:** 1 Telepsychiatry Research and Innovation Network Ltd Dhaka Bangladesh; 2 Department of Child, Adolescent, and Family Psychiatry National Institute of Mental Health Dhaka Bangladesh; 3 Sir Salimullah Medical College Hospital Dhaka Bangladesh

**Keywords:** domestic violence, COVID-19, mental health, violence, Bangladesh, lockdown, isolation, anxiety, stress, telemental health, telepsychiatry, web-based survey

## Abstract

**Background:**

The COVID-19 lockdown, the advent of working from home, and other unprecedent events have resulted in multilayer and multidimensional impacts on our personal, social, and occupational lives. Mental health conditions are deteriorating, financial crises are increasing in prevalence, and the need to stay at home has resulted in the increased prevalence of domestic violence. In Bangladesh, where domestic violence is already prevalent, the lockdown period and stay-at-home orders could result in more opportunities and increased scope for perpetrators of domestic violence.

**Objective:**

In this study, we aimed to determine the prevalence and pattern of domestic violence during the initial COVID-19 lockdown period in Bangladesh and the perceptions of domestic violence survivors with regard to mental health care.

**Methods:**

We conducted this cross-sectional web-based study among the Bangladeshi population and used a semistructured self-reported questionnaire to understand the patterns of domestic violence and perceptions on mental health care from August to September 2020. The questionnaire was disseminated on different organizational websites and social media pages (ie, those of organizations that provide mental health and domestic violence services). Data were analyzed by using IBM SPSS (version 22.0; IBM Corporation).

**Results:**

We found that 36.8% (50/136) of respondents had faced domestic violence at some point in their lives; psychological abuse was the most common type of violence. However, the prevalence of the economical abuse domestic violence type increased after the COVID-19 lockdown was enforced. Although 96.3% (102/136) of the participants believed that domestic violence survivors need mental health support, only 25% (34/136) of the respondents had an idea about the mental health services that are available for domestic violence survivors in Bangladesh and how and where they could avail mental health services.

**Conclusions:**

Domestic violence is one of the most well-known stressors that have direct impacts on physical and mental health. However, the burden of domestic violence is often underreported, and its impact on mental health is neglected in Bangladesh. The burden of this problem has increased during the COVID-19 crisis, and the cry for mental health support is obvious in the country. However, it is necessary to provide information about available support services; telepsychiatry can be good option for providing immediate mental health support in a convenient and cost-effective manner.

## Introduction

A person’s home is one of the safest and most secure and beloved areas where one can dream about enjoying every moment. However, a large number of people around the world feel insecure, frightened, and panicky due to experiencing physical, sexual, or psychological violence in their home environment (ie, violence from a familiar and related person). Domestic violence refers to violence that takes place in intimate relationships or when there is a relationship between the violence survivor and the perpetrator. This means that partners, ex-partners, close or distant family members, relatives, or family friends—anyone—can cause such violence. According to the Domestic Violence Prevention and Protection Act 2010, domestic violence is defined as “physical, psychological, sexual or economic abuse against a woman or child of a family by any other person of that family with whom the victim is, or has been, in family relationship.” Domestic violence, which is also sometimes referred to as intimate partner violence, is normally assumed to be perpetrated against females, but in general, domestic violence can be perpetrated against anyone in an intimate relationship. The main determinants of these kinds of behaviors may be the desire to acquire or establish a power balance and exert control over a partner or relationship. Usually, the unequal dynamics in a relation are thought to be one of the main contributing factors [1].

The World Health Organization has estimated that about 35% of women worldwide have experienced either physical and/or sexual intimate partner violence or nonpartner sexual violence in their lifetime [2]. Although domestic violence is a universal problem, sociocultural influences and the portrayal of domestic violence in the media characterize its local pattern and define the acceptance, expression, and explanation of the problem. According to the Bangladesh Bureau of Statistics, more than 70% of married women in Bangladesh have reported at least 1 physical or sexual violence incident in their conjugal life [3]. There are many unreported incidents of physical, emotional, verbal, sexual, and financial abuse. The recent COVID-19 pandemic has made gender-based violence more prominent, as the problem has deepened and has invaded new families [4]. This global health crisis has imposed multidimensional and multiphase negative impacts on health, social areas, and economic sectors. Researchers have found that gender-based violence increases during and after unprecedented humanitarian crises, including conflicts and natural disasters [4].

There are several determinants that can result in the increased prevalence of gender-based violence or domestic violence during any emergency or crisis. First, the preexisting economic dependency of females on their male counterparts intensifies the risk of gender-based violence during days of crisis [5]. Second, during a crisis situation, survivors of gender-based violence are often deprived of ample legal support, and as a result, the perpetrators remain unpunished [6]. Third, the absence of scrutiny from the outside world during a pandemic can distort the power balance at home, which can result in violence and abuse [7].

The United Nations Fund for Population Activities reported at least a 20% increase in the incidence of violence during the COVID-19 pandemic in 193 member states of the United Nations [8]. A telesurvey, which was conducted by the Manusher Jonno Foundation in 53 districts of Bangladesh, revealed that 9844 women and 2896 children experienced domestic violence until June 2020. Further, of the total number of reported cases, 4160 participants experienced such violence for the first time in their lives [5].

The COVID-19 pandemic has brought unforeseen crises such as poverty, physical distancing, and violence to the surface, and a large number of people are losing jobs or having their salary cut. Many women earn money by working as helping hands for different families in Bangladesh, and this type of daily work is the only source of income for thousands of women. However, when the daily number of people with SARS-CoV-2 infection increased, many families stopped allowing women to work in their homes due to the risk of infection. As a result, most women have started searching for food at one point or another and have become a vulnerable group that has a high risk of experiencing sexual abuse and violence, as evidenced by a couple of rape incidents that have been documented [6].

Domestic violence has resulted in long-term psychological trauma and impacts in addition to physical injuries and economic harassment. However, its threat to mental health and well-being is far more pervasive, severe, and long-term. Studies have shown that women exposed to gender-based violence have more than a twofold higher risk of developing a common mental disorder, including depression, anxiety disorder, posttraumatic stress disorder, and substance abuse, and suicidal tendencies [7]. A study conducted in Bangladesh found that reported negative health consequences range from simple injuries to grievous hurt and include psychological consequences, such as depression, anxiety, obsession, posttraumatic stress disorder, and even suicidal tendencies [9,10]. There has been an exponential rise in the prevalence of mental illnesses, including depression, anxiety, posttraumatic stress disorder, and suicidal ideation, among women who have experienced violence. Further, as the prevalence of violence against women increases, the need for mental health care also increases [11]. Moreover, there is a bidirectional relationship between mental health and gender-based violence. Women living with a severe mental illness are significantly more likely to experience violence [12]. Therefore, mental health care is an important and integral part of reducing the burden of gender-based violence. Globally, women have limited access to mental health care, and the situation is more disappointing in low- and middle-income countries like Bangladesh. In this country, 1 psychiatrist serves roughly 1 million people, and there are less than 50 clinical psychologists among the whole population [13]. The situation becomes more complicated when one considers that most of the mental health professionals and mental health care facilities in Bangladesh are located in the capital city—Dhaka.

The main purpose of this study was to obtain a view of the rates of domestic violence during the pandemic and the perceptions of domestic violence survivors with regard to mental health care.

## Methods

### Study Summary

This cross-sectional study was conducted via a web-based platform from August to September 2020, which was when all of Bangladesh was struggling to manage the COVID-19 crisis. Both males and females aged above 16 years were eligible to participate in this study. A structured questionnaire was designed by the authors to fulfill the objectives of this study. Before participants’ participated in this study, an information sheet and a consent form were made available (ie, on the first page of the questionnaire). They were written in the Bangla languages so that information could be easily understood by all of the participants. The participants were free to withdraw at any time without having to provide explanations and were also allowed to not answer any questions if they wished. Moreover, personal identification information was not requested from participants to maintain information confidentiality.

### Design

The convenience and snowball sampling techniques were used to obtain the desired sample size. We used professional organizations’ and volunteer organizations’ social media pages to disseminate the questionnaire. In addition, we encouraged the recipients of the questionnaire to send the questionnaire to their friends and family members for completion. The questionnaire was composed of information on the sociodemographic characteristics of the respondents, the impact that the COVID-19 pandemic had on their personal lives, and domestic violence. Moreover, we also made efforts to determine the impact that domestic violence has on mental health and analyze the perceptions of respondents with regard to mental health help seeking.

### Data Collection

Although there were more than 35,000 people in the participating groups and organizations, a total of only 136 individuals voluntarily participated in this study by filling in the web-based questionnaire. We assumed that at least 1% of the questionnaire recipients would be interested in participating in this study and were expecting to have more than 350 respondents. However, we had a much lower response rate than what we initially assumed. Sociocultural stigma and the burden of the increased number of web-based surveys being conducted during the study period by different organizations might have had an impact on the response rate.

### Data Analysis Process

All information was gathered via Google Forms and was recoded into variables. The data were reviewed and sorted before starting the analysis. Data were analyzed by using IBM SPSS (version 22.0; IBM Corporation). The analysis techniques conducted were mainly for analyzing descriptive statistics.

### Ethical Consideration

This study was approved by the National Institute of Mental Health, Dhaka, and conformed to the provisions of the Declaration of Helsinki. All ethical procedures were maintained and followed during this study, including the process of maintaining web-based data privacy and security for sensitive data.

## Results

We analyzed 136 respondents aged between 17 and 50 years; their mean age was 24.26 years (SD 5.15 years). The sociodemographic characteristics of the respondents are reported in Table 1.

A total of 36.8% (50/136) of the respondents reported that they were survivors of domestic violence, and this prevalence rate was about 3 times higher among females (36/50, 72%) than that among males (14/50, 28%). Of the 50 domestic violence survivors, 16 (32%) experienced domestic violence very often, 10 (20%) sometimes experienced domestic violence, and 24 (48%) rarely experienced domestic violence. The most common type of violence that the respondents faced was mental abuse (n=34, 65.4%; Table 2). However, one should also consider that 3.7% of the respondents were unwilling to answer the question regarding whether they were survivors of domestic violence. Moreover, 24.2% (33/136) of respondents (male: 21.2%; female: 78.8%) experienced domestic violence for the first time during the lockdown period, and this violence was perpetrated by their family members. Further, 5.1% (n=7) of domestic violence survivors faced such violence very often during the lockdown period. In addition, 22.8% (31/136) of the participants revealed that their other family members also experienced domestic violence, and 37.5% (51/136) of respondents came to know that other relatives or friends experienced domestic violence during the lockdown period.

In total, 41.2% of participants reported that they faced a mild economic crisis, and 30.1% of participants reported that they faced a significant economic crisis. Table 2 shows that among the different forms of abuse, the prevalence of economical abuse almost tripled during the lockdown period. Furthermore, a positive correlation was observed between changes in economic conditions after the COVID-19 lockdown started and experiencing domestic violence for the first time after the COVID-19 pandemic started (n=134; *r*=0.107); however, the correlation was not statistically significant (*P*=.89).

The participants had different opinions regarding the reasons behind domestic violence during the COVID-19 pandemic. These opinions are shown in Table 3.

Our study also revealed that about 45% of the respondents experienced web-based sexual harassment in their lifetime. We also found that social media engagement was linked to respondents’ experiences of domestic violence. More than 96% (102/136, 96.3%) of the respondents believed that they needed mental health support. However, 75% (102/136) of the respondents did not know how to avail such services in Bangladesh.

**Table 1:** Sociodemographic characteristics of the respondents (N=136).

Characteristics		Respondents, n (%)
**Gender**		
	Male	35 (25.7)
	Female	101 (74.3)
**Marital status**		
	Unmarried	103 (75.7)
	Married	30 (22.1)
	Separated	2 (1.5)
	Widowed	1 (0.7)
**Occupation**		
	Student	92 (67.6)
	Service holder	26 (19.1)
	Businessperson	5 (3.7)
	Unemployed	8 (5.9)
	Other	5 (3.7)
**Family status**		
	Nuclear family	110 (80.9)
	Joint family	20 (14.7)
	Currently staying out of family	6 (4.4)

**Table 2:** Distribution of the different types of violence before the lockdown period and after the lockdown was imposed.

Types of violence	Before lockdown, n (%)	After the first lockdown was imposed, n (%)
Physical	10 (19.2)	4 (10.5)
Psychological	34 (65.2)	26 (68.4)
Sexual	1 (1.9)	1 (2.6)
Economical	3 (5.8)	6 (15.8)
Other harmful traditional practices	4 (7.7)	1 (2.6)

**Table 3:** The possible reasons behind the increased prevalence of domestic violence.

Reasons	Participants, n (%)
Failure to manage increasing stress	35 (25.7)
Deterioration of economic status	26 (19.1)
Increased duration of stay	26 (19.1)
Fear of getting infected and panic	17 (12.5)
Moral decay and family learning	12 (8.8)
Patriarchy	10 (7.4)
Deteriorating mental health and preexisting mental illness	7 (5.1)
Other	3 (2.2)

## Discussion

Violence against women is a major public health issue, and managing this issue is a key objective of the sustainable development goals of Bangladesh. However, during the COVID-19 crisis, the incidence of domestic violence has supposedly increased, and China, France, Italy, Brazil, and Spain have reported the increased incidence of domestic violence during the COVID-19 crisis [14]. There is a significantly higher number of females reporting that they have experienced domestic violence compared to this number among males. A male-dominated society or patriarchal society, like that in Bangladesh, provides more scope for females to experience domestic violence [1]. Other factors that participants reported as the causes of violence were financial instability, mental stress, and the increased number of opportunities resulting from the COVID-19 lockdown. Moreover, it has also been observed that pandemics and any other crisis situations increase the risk of abuse among vulnerable populations [8]. However, the number of males reporting to be survivors of domestic violence is also noteworthy for a male-dominated country like Bangladesh, and this topic demands further research.

The COVID-19 pandemic has increased the risk of domestic violence, as survivors have had to remain with their abusive family members for a longer period of time [15,16]. It has been observed that mothers-in-law and sisters-in-law act as instigators in domestic violence incidents in Bangladesh, and this finding is consistent with those of other qualitative studies in India [17,18]. Therefore, the COVID-19 lockdown has provided more opportunities for intimate partners and other abusive family members to engage in violent activities. As those who experience domestic violence are at risk of developing various mental and physical conditions [19], a responsive reporting system, along with effective mental health care, is of utmost importance to domestic violence survivors [20]. Moreover, it is necessary to inform the survivors of domestic violence about the available mental health support services in a country. Even if these survivors know about the different hospitals in a country, they fail to avail such services due to geographic distance, the lack of freedom to seek such services, the fear of experiencing further abuse from their families, and the need to provide the cost of such services. These barriers can be minimized by telepsychiatry or e-mental health services. Telepsychiatry (ie, the long-distance provision of psychiatry services) emerged with the promising effect of overcoming potential barriers and improving access to mental health services for all [19,21].

The role of telepsychiatry has become more important during the COVID-19 crisis in Bangladesh, as telepsychiatry services provide psychological support to the those who experience gender-based violence. Telepsychiatry support can provide more anonymity by providing the option of communicating with professionals 24-7 through audio, video, or chat platforms, thereby ensuring that that the limited number of professionals serve the highest possible number of people. Domestic violence survivors need to have the phone numbers of national call centers to obtain different kinds of support. They also need different web-based and mobile-based mental health services, such as MonerDaktar [22], MindTale, and Maya Apa; these services have made remarkable contributions by providing mental health support. Governments and policy makers should consider how they can use technology-based psychiatry services or digital psychiatry services to serve the thousands of women who are facing different forms of violence every day and need mental health support.

### Conclusion

The prevalence of domestic violence has been increasing in Bangladesh during the COVID-19 crisis, and this crisis will persist for a few more months or years. As such, mental health burden resulting from domestic violence will rise in prevalence. We need a multilayered holistic plan for supporting and providing cost-effective, high-quality mental health support for domestic violence survivors.
